# Development and implementation of measurement-based care for children and youth with complex mental and neurodevelopmental needs: early experiences from Ontario’s Extensive Needs Service

**DOI:** 10.3389/fpsyt.2025.1704325

**Published:** 2026-01-05

**Authors:** Victoria Rombos, Zainab O. Salami, Genevieve Ferguson, Renee Baysarowich, Kathryn Decker, Nicholas Denomey, Irene Drmic, Jordan Edwards, Lamia Hayawi, Thiyaana Jeyabalan, Taylor Johansen, Toni Lui, Karen Margallo, Tamara Milicevic, Nicholas Mitsakakis, Stephanie Sutherland, Amedeo D’Angiulli, Richard Webster, Melanie Penner

**Affiliations:** 1 Holland Bloorview Kids Rehabilitation Hospital, Toronto, ON, Canada; 2 Children’s Hospital of Eastern Ontario, Ottawa, ON, Canada; 3 Hamilton Health Sciences, Hamilton, ON, Canada

**Keywords:** neurodevelopmental disorders, mental disorders, behavioral symptoms, mental health services, measurement-based care (MBC)

## Abstract

**Introduction:**

Measurement-based care (MBC) is a structured approach to collecting patient-reported outcome measures (PROMs) to inform clinical care. While MBC is routinely used in mental health settings, its application in neurodevelopmental populations—particularly those with co-occurring physical and mental health conditions—remains limited.

**Methods:**

MBC was implemented across three Ontario hospital Extensive Needs Service (ENS) sites in Toronto, Hamilton, and Ottawa. Selection of PROMs was informed by a focused literature review and consultation with clinicians, researchers, and family advisors. A logic model was developed to guide evaluation and link measures to anticipated outcomes. Clinicians received training and support to integrate PROMs into clinical workflows. Data were collected at baseline and at regular intervals.

**Results:**

Between April 2023 and April 2025, 381 participants entered the ENS program, and 36 were discharged. Their duration of participation ranged from 3 to 20 months. Each site engaged clinical staff in PROM completion. Completion rates for measures were higher at baseline and declined over time. Shorter PROMs showed higher completion rates compared to longer ones.

**Discussion:**

This novel implementation of MBC provides important insights for MBC in pediatric populations with high complexity. Early and ongoing engagement of both clinicians and families is important to success, which is also closely tied to the degree to which MBC is integrated into care processes. MBC remains necessary to guide clinical care and treatment planning for children with complex, intersecting needs. It is also helpful when evaluating new programs and generating foundational evidence on the effectiveness of therapies for this population.

## 1 Introduction

Measurement-based care (MBC) is a systematic approach to collecting outcome measures either before or during clinical visits to inform patient care (1), and to facilitate improvement and evaluation within clinical programs (2). Tracking growth parameters, vital signs, and laboratory markers is routine practice in many medical clinics; however, judging baseline needs, therapeutic progress, and intervention options in neurodevelopmental, behavioral, and mental health clinics more often relies on reports from children/youth and their family caregivers (1). As such, MBC in neurodevelopmental clinical settings is predominantly based on patient-/parent-reported outcome measures (PROMs). Routine implementation of PROMs using MBC strategies has been shown to improve individual patient outcomes (2), including for those who have not responded to first-line treatments (3). Beyond this impact on individual outcomes, the harmonization of outcome measures can support the development of a learning health system (4, 5), whereby analysis of outcomes across patients can be used for timely evaluation of care models and the generation of new knowledge using traditional research approaches.

Children/youth with neurodevelopmental conditions (such as autism, intellectual disability, and attention-deficit/hyperactivity disorder [ADHD]), particularly those with co-occurring mental health and/or physical health conditions, sometimes experience challenges in achieving meaningful therapeutic responses with available services (6–8). Often, these children/youth have difficulty accessing adequate community-based care (9) and can have disproportionately high use of acute care, such as emergency departments and inpatient admissions, compared to neurotypical populations (10–12). There is an urgent need to understand which treatment modalities work for which children/youth and the resulting impact in mitigating negative outcomes, such as potentially avoidable acute care visits. Given the unique needs of this population and the necessity of tailored approaches, MBC allows clinicians to evaluate and modify care in real time using data reported by the people who know them best. Coordinated implementation of MBC offers one approach to evaluate care models and their resulting impacts across a variety of domains, including service use and improved outcomes for patients.

To our knowledge, there has been little published research exploring MBC in neurodevelopmental populations to date, with comparatively more work coming from mental health clinics (1, 13, 14). One exception is the Autism Care Network (previously named the Autism Learning Health Network) (15). The aims of this North American network were to reduce variations in care across member sites and emphasize best practices, with a focus on inclusion of previously underrepresented groups. Unfortunately, this network focused only on one condition (autism), and there are no further (or longitudinal) results published from it. The implementation of longitudinal MBC is key to generating much-needed data to inform treatment modalities for children/youth with combined neurodevelopmental, behavioral, and mental health conditions—particularly those who have not responded to first-line therapies—as well as to inform supports for caregivers/families. The objective of this work was to describe the development and initial implementation of MBC in the Extensive Needs Service (ENS), a novel program funded by the Ontario government to provide wraparound care to children/youth with intersecting developmental, physical, mental health, and social complexities.

## 2 Methods

While the outcomes presented here do not include any personal health information, each site still obtained research ethics approval, including opt-out/waiver of consent for data collection.

### 2.1 Clinical setting

The ENS is a dedicated pathway offering specialized services for children and youth with complex, unmet needs in Ontario to promote optimal outcomes (16). The service is a client-centered program structured around interdisciplinary, trauma-informed, accessible care tailored to each client’s needs. Specialized, wraparound services in ENS are jointly funded through Ontario Health via the Ministry of Health (MOH) and the Ministry of Children, Communities and Social Services (MCCSS), and are delivered through specialized centers able to provide continuity of care throughout childhood and adolescence. Three Ontario hospitals were funded as part of a 3-year proof of concept of the ENS program: Holland Bloorview Kids Rehabilitation Hospital (HB), McMaster Children’s Hospital (MCH), and the Children’s Hospital of Eastern Ontario (CHEO).

Clients access ENS through various referral pathways, including external community providers (e.g., physicians, education professionals, child welfare agencies), internal providers (i.e., cross-departmental referrals within the hospital), and, in some regions, family self-referral. After a referral is received, the referral source and/or family caregivers and, when relevant, the child/youth are contacted to gather more information to help inform next steps. During this session, ENS team members collect preliminary information to inform initial service provision, as well as any further information required to determine eligibility. Referred children/youth are subsequently discussed at ENS team meetings for preliminary treatment planning. The exact complement of service providers differs across ENS sites; however, services generally include specialized behavior services, mental health assessment and treatment, social work, care coordination, occupational therapy, speech-language pathology, medication management, respite (or assistance accessing community respite), and transition support to community service providers. ENS is not strictly time-limited, and discharge is based on several factors, including the child/youth’s progress, goal attainment, and overall readiness to access community supports/programs. Outcome measures are completed at baseline and throughout the program and will be described in further detail below.

### 2.2 Participants

Inclusion criteria for the program include: i) age up to 18 years; ii) co-occurring neurodevelopmental conditions or an acquired brain injury and mental health condition(s), as well as possible chronic physical health condition(s); iii) existing needs that are not currently met with respect to: a) challenging/interfering behaviors (lasting at least 12 months or escalating over the past 6 months); b) having already accessed several healthcare/service providers across sectors and/or being unable to engage in services due to extraordinary circumstances within the family system; and c) safety concerns that are a barrier to participation in home, school, or community settings. Caregiver/family complexity is also considered, informing unmet needs of the family unit.

### 2.3 Implementation of MBC in ENS

Here we describe the initial phases of implementation of MBC within ENS, including the identification of measures, consultation with the clinical team, development of the logic model, and pilot implementation.

#### 2.3.1 Focused literature review

Given the timely nature of establishing the Extensive Needs Service across three sites in Ontario, the research team undertook focused literature reviews in two areas:

- identifying similar programs to determine their care models and evaluation strategies; and
- identifying measures that would capture each key program aim.

Program aims were co-developed with leadership from both funding ministries (MOH/MCCSS). One team member conducted the literature search and filtered results. The search was conducted using MEDLINE, PubMed, CINAHL, and PsycINFO databases from 3–13 February 2023, with articles from 2008 onward. Search terms included the following: fragility; fragility of child; fragility of family; vulnerable populations; complexity; complexness; co-occurring conditions; developmental complexities; social vulnerabilities; evidence-based treatment; trauma-informed treatment; measurement-based care; unmet needs; behavior/symptom improvement; high-intensity user. English-language peer-reviewed studies were included.

Five programs akin to the ENS vision were identified in the United States, United Kingdom, and Australia. These interventions used a combination of quantitative and qualitative methods to evaluate their programs, including both pre- and post-measurement-based tools (17), and interviews with families and clients (18–20). When reviewing program models, the Access to Tailored Autism Integrated Care protocol, implemented in California, United States, highlighted the success of having an identified clinical champion who connected with the research team on a biweekly basis to evaluate implementation of the program (19). The Children and Young People’s Health Partnership Evelina London model emphasized the importance of incorporating family perspectives early on to determine the key components considered essential for effective integrated care services for children and families (21). This was exemplified when families described “integrated service as providing holistic care, within a family,” highlighting the patient’s nurse and pediatrician discussing the care plan together.

Through a combination of literature review, consultation with adjacent programs, and discussion with ENS clinicians, we identified multiple candidate measures related to clinical symptom improvement across mental health and behavioral presentations: the Child Behavior Checklist (22), the Behavioral Assessment System for Children (3^rd^ edition) (23), and the Aberrant Behavior Checklist (24). Quality of life measures (25, 26) were added as an additional way to assess clinical symptoms and unmet needs. Measures of adaptive skills (i.e., daily functioning) were included to further understand client needs. To measure family distress levels, the Brief Family Distress Scale (BFDS) (27) was identified to capture fragility at the family level. The Resource Use Questionnaire (RUQ) was identified to measure resource use in autistic children (28); this was modified with permission from the authors to capture a transdiagnostic clinical group. Once possible measures for each domain were identified, we reviewed the list and began narrowing it down based on relevance, sensitivity to change, inclusive language, and completion burden (see **Appendix 1**).

#### 2.3.2 Consultation with ENS clinicians

Findings from the reviews were presented by the research team to the initial members of the ENS Research and Evaluation Working Group, which consisted of a project manager from CHEO; a program director, clinical manager, clinical/research lead, behavior therapists, and social workers from HB; and a program director, clinical/local research lead, researcher, data research coordinator, and various clinicians (e.g., psychologists, social workers, psychiatrist, behavior therapist) from MCH. CHEO also consulted with its Neurodevelopmental Health Patient and Family Advisory Committee (NDH-PFAC) at this stage. At this time, ENS clinicians suggested that a standardized way of setting treatment goals and measuring progress would be beneficial. Findings were subsequently presented to the ENS clinical working group, whose membership included clinicians and operational leads across the three sites. Working group members reviewed the included tools at the item level and flagged language perceived as overly pathologizing, particularly in relation to behavior. Candidate measures were also reviewed for relevance and anticipated completion burden. Certain aspects of the RUQ were deemed important to measure on a quarterly basis, including acute care usage (emergency department visits and inpatient admissions), interventions from law enforcement or paramedics, missed school days, and parent/caregiver missed workdays. Together, the ENS clinical and evaluation working groups determined that the measurement battery would be applied to ENS clients receiving the interdisciplinary wraparound component of ENS (i.e., multiple types of services through the program) with an expected treatment duration of at least 6 months.

#### 2.3.3 Evaluation approach

**Figure 1:**
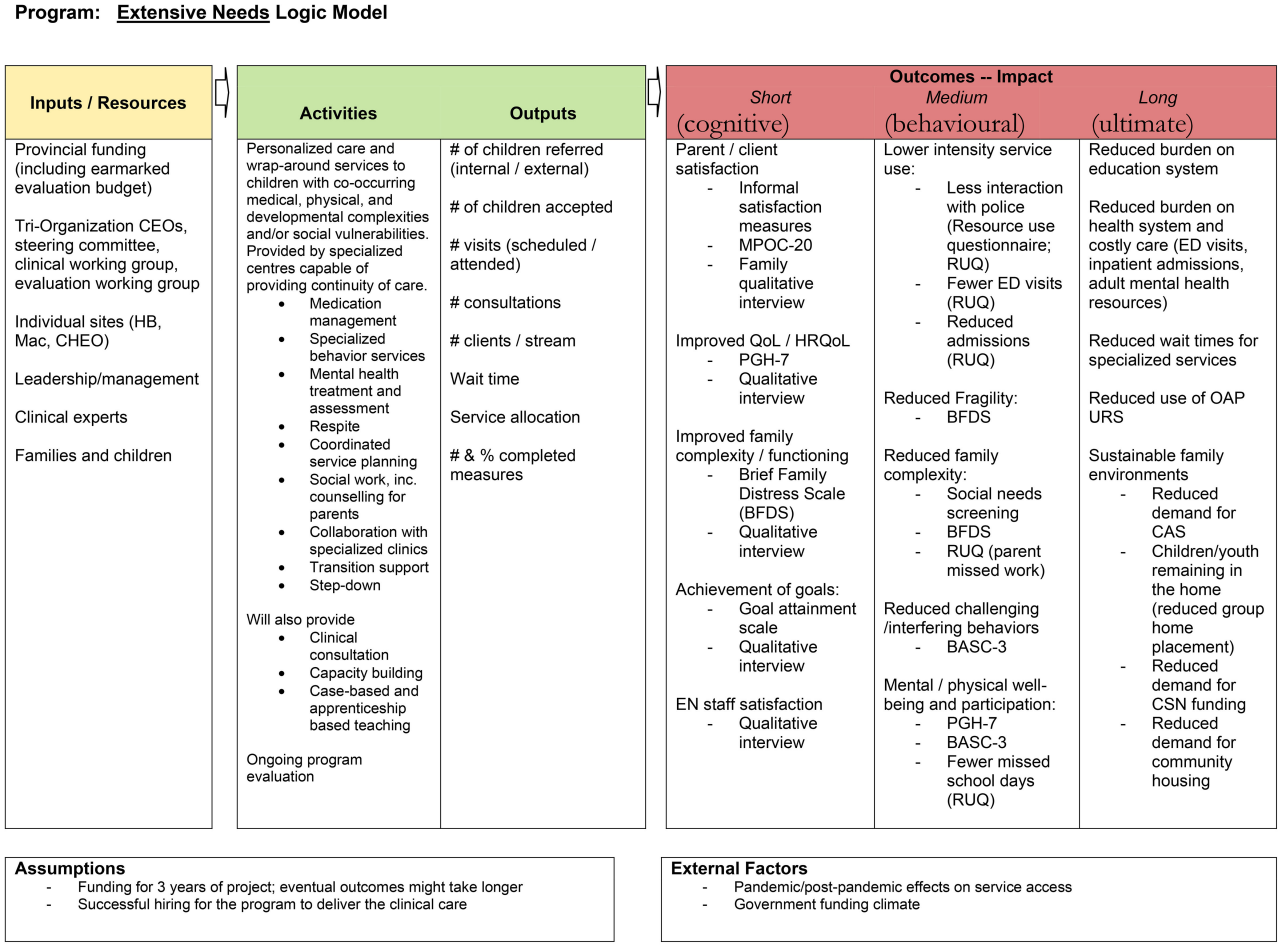
Logic model. CEO, Chief executive officer; MPOC-20, Measures of Processes of Care; QoL, Quality of life; HRQoL, Health-related quality of life; PGH-7, Pediatric Global Health Measure; ED. Emergency department; BASC-3, Behavior Assessment System for Children; OAP URS, Ontario Autism Program Urgent Response Service; CAS, Children’s Aid Services; CSN, Complex Special Needs.

Based on the results of the focused literature review and initial identification of potential clinically relevant PROMs, we developed an evaluation framework and logic model (**Figure 1**) to organize and contextualize the broader program evaluation. Logic models have been successful in fields such as program planning, implementation, and primary care and are useful in “providing a common approach to integrating planning, implementation, and evaluation” (54). The logic model allowed our team to link the measures with their corresponding clinical domains (high-intensity users, complexity, fragility, unmet needs, and clinical symptom improvement) and create a visual representation of anticipated short-, medium-, and long-term outcomes (55). This helped inform the shared mission, vision, and objectives of the evaluation (56).

#### 2.3.4 Pilot phase of outcome measures

During the pilot phase, which lasted 1 month, the evaluation team first introduced the PROMs to the ENS clinical team, leading training sessions and providing other opportunities to review the measures prior to using them with clients and families. These training sessions helped clinicians feel more prepared if caregivers had questions about any of the measures or if families requested support while completing them. Where available, standardized tools were accessed through their respective online platforms. The Pediatric Global Health measure (PGH-7) (25) and BFDS were built into REDCap so that these PROMs could also be completed by families online. Families were sent PROMs monthly via REDCap to complete independently or with support from a clinician or a member of the evaluation team, if required, in real time, either in person, virtually, or by phone. When required, interpreters were available so that non-English-speaking families could complete the PROMs. Evaluation team members at HB and CHEO built two of the measures (BFDS and RUQ) into the hospital electronic medical record (EMR), making it easier for clinicians to enter these data directly into the EMR during clinical intake interviews.

Using REDCap to collect PROMs made it easier for the evaluation team to create graphics and visuals to share with clinicians during weekly rounds. Pairing PROMs with clinical data helped the team generate a more fulsome report on each client’s progress and determine next steps and recommendations (57).

#### 2.3.5 Refinement of outcome measure battery and logic model

**Figure 2:**
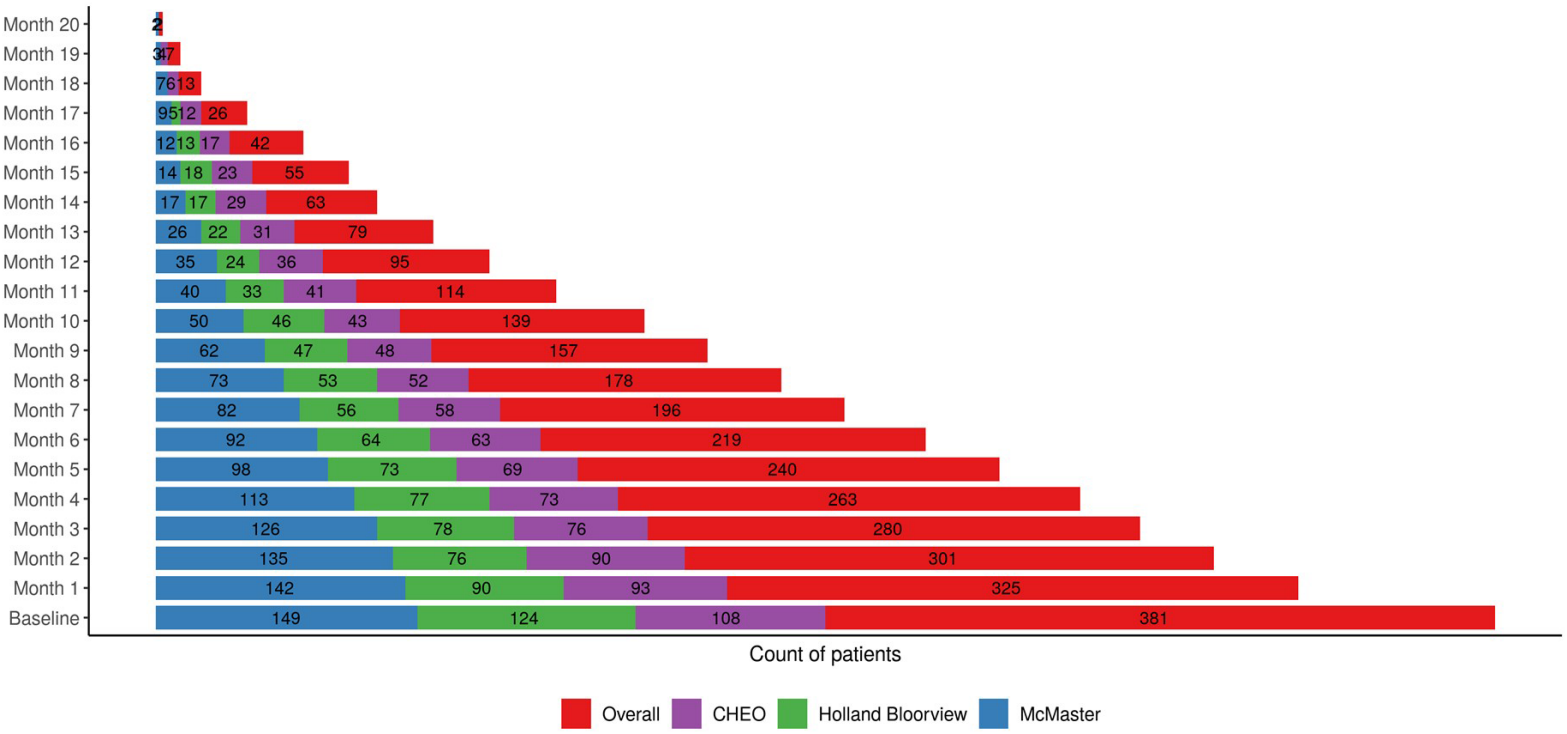
Total number of ENS clients. This figure represents the total number of clients at each monthly timepoint in the Extensive Needs Service. It represents how client numbers can fluctuate at varying durations of service.

**Figure 3:**
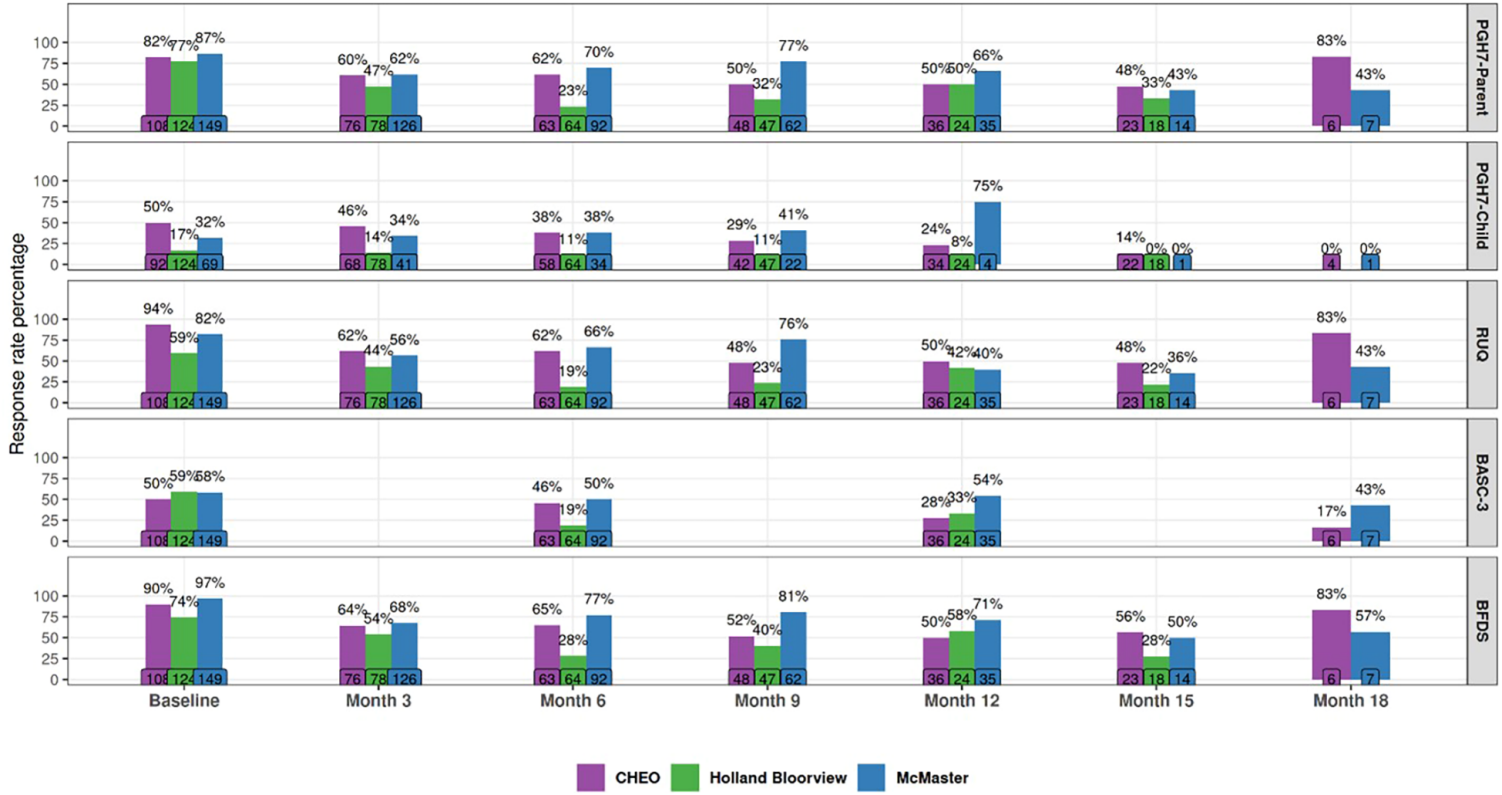
BFDS, PGH-7, RUQ & BASC-3 Completion rates (Quarterly). This figure shows the response rate for all measures included in the battery across the Tri-Organization sites (Holland Bloorview, McMaster Children’s Hospital, and Children’s Hospital of Eastern Ontario. Response rates are calculated every 3 months here, apart from the BASC-3, which is administered every 6 months. The PGH-7 Child is completed only when the child is able to answer the questions independently.

Pilot experiences were brought back to the ENS Evaluation Working Group for review and discussion. Completion of longer tools (ABAS-3, BASC-3) was consistently identified as a challenge. The Working Group noted that the BASC-3 included an Adaptive Skills subscale, which could be redundant with the ABAS-3. For this reason, the ABAS-3 was removed from the battery. The program logic model was updated accordingly. Completion rates of measures were collected at the site level and compiled across tools and sites for the BFDS, RUQ, BASC-3, and PGH-7. The GAS was implemented later than the other tools and will not be presented here. Details of the final battery of outcome measures are available in **Table 1**. The data presented below (**Figures 2**, **3**) represent PROM completion rates from April 2023 to April 2025.

**Table 1:** Extensive needs service – measures.

Categories	Measures	Family report and/or youth report?	REDCap, interview or other?*	Cost per use?	Time to complete	Time point(s)
Family distress level	*The Brief Family Distress Scale* (BFDS)– 1 item (rating scale) Weiss, J. A., & Lunsky, Y. (27).	Family	REDCap	No	1–5 min	Baseline, monthly
Quality of life	*PROMIS Pediatric Global Health Measure (PGH-7)* • Ages 8-17 (self-report), ages 5-17 (parent report) - 7 items (rating scale) Forrest et al. (25)	Family and self-report	REDCap	No	1–5 min	Baseline, q3months, end of service
Symptomatic/behaviour	*Behavior Assessment System for Children (BASC-3)* • Parent rating scales (PRS)-child, ages 6-11–175 items (rating scale) • Parent rating scales (PRS)–adolescent, ages 12-21, - 173 items (rating scale) • Self-report of personality (SRP)-child, ages 8-11–137 items (rating scale)_ • Self-report of personality (SRP)–adolescent, ages 12-21–189 items (rating scale) Reynolds, C.R. & Kamphaus, R.W. (23).	Parent and Self-report	QGlobal or paper copy	Yes	30–60 min	Baseline, q6 months, end of service
Goal-setting	*Goal Attainment Scale (GAS)* - open goal chart with areas for family/youth to identify goals & define levels Kiresuk, T.J., et al., 1994. (58)	Family and self-report	Clinician interview	No	10–15 mins	Baseline, revisiting throughout, end of service/treatment
Resource-use	*Resource Use Questionnaire (RUQ)* – revised for EN (with permission from author) • Mixed format *RUQ – Quarterly* Includes: emergency services, ED visits, admissions, missed school days, missed work days (parent/guardian) Ungar, W. J., et al. (28)	Family report Family report	Combined with intake form, completed by clinician REDCap q3m to capture salient items	No	15–30 mins	Baseline, end of service or annually (whichever is shorter) REDCap q3m to capture salient items

REDCap - (Research Electronic Data Capture) is a secure web application for building and managing online surveys and databases; PROMIS^®^ - (Patient-Reported Outcomes Measurement Information System) is a set of person-centered measures that evaluates and monitors physical, mental, and social health in adults and children; PRS , Parent rating scales; SRP, Self-report; Q-global, Pearson’s web-based system for administering, scoring and reporting Pearson assessments; q3m, indicates the measure is administered quarterly; q6m, indicates the measure is administered every 6 months.

Completion rates were calculated by dividing the number of participants who completed each measure at a specific time point (numerator) by the total number of participants expected to be at that time point (denominator). The denominator was determined based on each participant’s service start date (i.e., when they completed their initial evaluation assessments). Participants were then tracked over time and included in the denominator for each time point based on their service start date. If a participant was discharged, they were included in the denominator only for the period during which they were enrolled in the program.

## 3 Results

**Figure 4:**
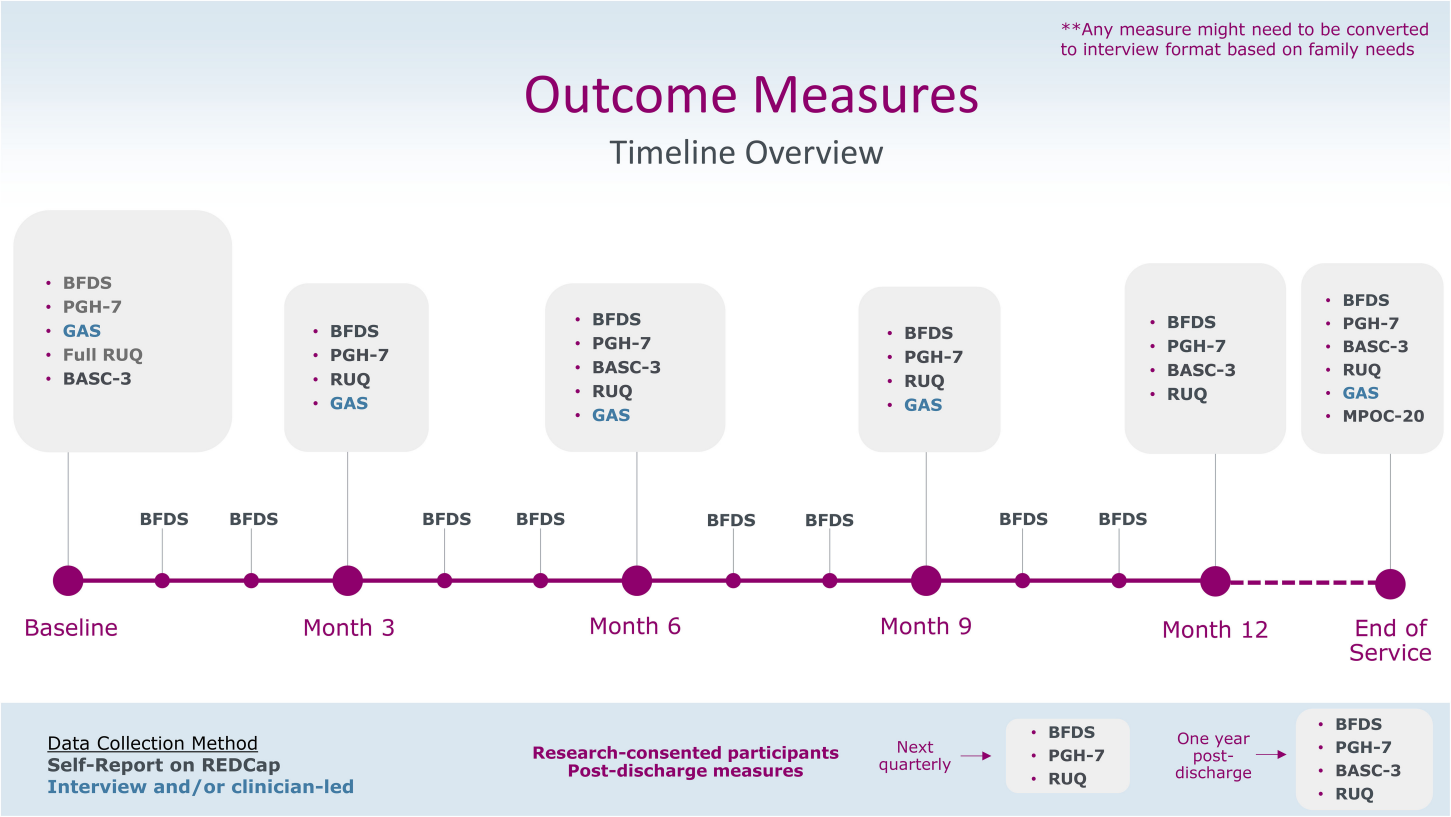
Outcome measures timeline. BFDS, Brief Family Distress Scale; PGH-7, Pediatric Global Health Measure; GAS, Goal Attainment Scale; RUQ, Resource Use Questionnaire; BASC-3, Behavior Assessment System for Children; MPOC-20, Measures of Processes of Care; REDCap, (Research Electronic Data Capture) is a secure web application for building and managing online surveys and databases.

The ENS MBC logic model is presented in **Figure 1**, and the streamlined outcome measure battery and measurement time points is available in **Figure 4**. Implementation of outcome measures in ENS began on 27 April 2023 and is ongoing.

### 3.1 HB site implementation

We built our program based on the primary clinician model adopted as part of ENS at HB. In this model, the main clinician working with the client is identified as their “primary clinician” and acts as the main point of contact for the client’s care (59). This clinician is then responsible for ensuring that the client’s PROMs are completed. The evaluation team requested feedback from clinicians and received comments highlighting both benefits and drawbacks of their experiences. Families reported that it was helpful to have ENS clinicians or program evaluation staff support them in completing the measures, with many expressing concerns about managing PROM completion on their own.

In the initial program delivery at HB, the ENS team would decide on the complement of services that the child would receive. The first available clinician from this team would see the child and their family, triggering the initiation of measure completion, and other services would join as available. In practice, some services took longer to start, and families provided feedback that they still felt as though they were waiting for aspects of ENS. Based on this feedback, the model recently changed so that ENS begins only once all services are ready to start with the child and their family.

### 3.2 CHEO site implementation

CHEO initially followed a process similar to HB but has transitioned to a streamlined start model, in which families begin once a “pod” clinical team—consisting of a behavioral analyst, service planning coordinator, and social worker—is available to support the interdisciplinary assessment with families entering ENS. The ENS outcome measures are fully clinically integrated, with measures completed live with families and data inputted directly into CHEO’s EMR (EPIC). Once this assessment is completed, the clinical team begins to enlist additional members based on family needs and goals. These additions may include a respite worker, child and youth counselor, service planning coordinator, behavior therapists, and members of the medical team (i.e., occupational therapist, speech-language pathologist, registered nurse, registered dietitian, or psychologist). Team members are responsible for conducting additional clinically relevant assessments that support development of ENS goals, the treatment plan, and benchmarks for discharge criteria. Once the treatment plan has been established and agreed upon by the clinical team and family, the treatment phase begins, and subsequent outcome measures are completed with the client and their family. The program evaluation team remains actively involved in supporting clinicians with the use of outcome measures, including providing training on administration, interpretation, and use of tracking sheets. They also attend clinical rounds to offer real-time support, answer questions, and ensure clinicians feel confident and equipped throughout the data collection process.

### 3.3 MCH site implementation

At MCH, the family participates in a welcome meeting with the social worker and integrated service consultant, followed by initiation of a family-based assessment. More specifically, the Family Check-Up^®^ (FCU) (60, 61)—a brief, tailored, ecological intervention developed to decrease childhood emotional and behavioral challenges and improve caregiver well-being—is delivered to families when feasible and appropriate. Following the initial assessment, each client is presented to an interdisciplinary pod team consisting of the assigned social worker and integrated service consultant, as well as a behavior analyst, behavior therapist, additional social workers, speech-language pathologist, occupational therapist, dietitian, psychologist, and pediatrician, to support further assessment and treatment planning.

The integrated service consultant serves as the primary point of contact for the family in the program and is responsible for managing and ensuring the completion of outcome measures using REDCap. They use the platform to schedule follow-up assessments, share secure links with families to complete the outcome measures independently, and provide secure access to other care team members who may assist in the completion process. In most cases, the integrated service consultant directly supports the family in completing the outcome measures during appointments. In addition, they actively monitor the status of each measure within REDCap to track progress and identify whether any follow-up actions are required to ensure timely and complete data collection.

As shown in **Figure 3**, CHEO and MCH have a higher number of completed measures compared to HB. This may be related to the earlier clinical integration of PROMs at CHEO and MCH (i.e., a clinician is responsible for ensuring measure completion). Having measures delivered by a clinician and recorded directly into the EMR or REDCap increases accessibility for clinicians and families and decreases logistical complexity by avoiding paper-and-pencil administration or manual data entry (57). These are all factors that can influence completion rates for MBC. While HB had the measures built into REDCap, the lower completion rates suggest that families may be more successful when completing these measures with their primary clinician.

Clinicians across the Tri-Org requested additional support and training on how to co-create GAS goals and facilitate goal-setting conversations with clients and families. As a result, rollout of the GAS lagged behind other PROMs.

### 3.4 Completion of measures

Three hundred and eighty-one clients across sites (CHEO: 108, Holland Bloorview: 124, McMaster: 149) have entered the interdisciplinary intensive wraparound stream of ENS as of April 2025 (**Figure 2**). Based on the date range of April 2023 to April 2025, clients have been in ENS for varying durations, ranging from 3 months to 20 months. We see a decline in numbers over time because some clients have not yet reached later time points or were discharged prior to 20 months. This is noticeable at the 12-month mark, when the number of clients drops below 100 and continues to decrease over time. Thirty-six clients have been discharged from the program.

Completion rates have varied across sites, measures, and clinical time points (**Figure 3**). Baseline response rates were generally high across all measures, with sites reaching up to 97% for the BFDS, while rates were lower for the PGH-7 Child and the BASC-3. By Month 18, only the BFDS and PGH-7 Parent show notable retention (up to 83% at McMaster), while completion rates for other measures fall to very low levels (often below 20%).

Completion rates for the BFDS are provided in **Figure 3**. Other tools—including the PGH-7 (completed by parents for all clients and by clients themselves when able), the RUQ (full version at baseline and abbreviated version quarterly), and the BASC-3—are completed either every 3 or 6 months; completion of these tools is also reported in **Figure 3**.

## 4 Discussion

In this paper, we have detailed the design and implementation of MBC in an innovative new multi-site program, ENS, designed to meet the needs of children and families with intersecting developmental, behavioral, mental health, physical, and social complexities. Implementing MBC in the context of this program has involved careful consideration of its aims and target population, repeatedly seeking input from clinicians, and measuring progress during implementation. Because ENS represents a new, cross-sectoral program in the service landscape, MBC is essential for tracking individual client progress, as well as site-level and program-level achievements and challenges. Indeed, our initial results show that further improvements are needed to optimize completion of measures and integration of those measures with clinical activities. Our implementation has provided many instructive lessons for similar programs seeking to use MBC.

Our initial literature search identified few comparable programs using MBC, particularly when considering the transdiagnostic nature of ENS. Our group of measures aimed to capture wide-ranging program aims, including clinical symptom improvement, better quality of life, reduced reliance on acute care, and improved family well-being. Compared to other programs, one notable weakness of our approach was the lack of robust engagement of families and youth in the early planning stages (21). Now that the program is established and operating at full capacity, our team has a clearer understanding of the types of clients and families in ENS. Accordingly, we have recruited a lived experience advisory group specifically for the ENS evaluation, comprised of families who have participated in the program. We also regularly solicited feedback from clinicians about the measures and adapted the measure battery accordingly.

Looking across ENS sites, measurement completion rates are linked with the degree of family and clinician engagement. For example, lower completion rates helped us identify necessary changes to clinical service delivery at one of the sites, such as waiting until all indicated services were available so that the child and family experience true wraparound care and greater engagement with the program. Responsibility for ensuring completion of outcome measures has shifted from evaluation staff to program clinicians. Martin-Cook et al. (62) also identified the importance of clinician engagement in implementing MBC. They described key aspects to improve this engagement, including focusing on the impact of MBC on client care, working with clinicians to develop a list of barriers, and regularly meeting to address these. To emphasize the institutional benefits of MBC, Cooper et al. (63) included project status updates at monthly clinical division meetings as a way to keep staff engaged with the project and maintain momentum.

A variable that played a significant role in clinician engagement was training and ensuring that the team felt prepared and comfortable implementing MBC during their appointments. The evaluation team at HB scheduled several training sessions during the inception of ENS to ensure that this was part of onboarding for all program staff. As described by Childs and Connors (64), establishing training in MBC processes and mandating them for newly onboarded clinicians is essential. To make these trainings accessible, the evaluation team sent polls to determine preferred meeting times and delivery methods (in person or videoconference). Multiple sessions were held to accommodate vacation and sick days. During these trainings, the evaluation team provided an overview of the outcome measurement tools, including how long each tool takes to complete, validation information, and common methods for using the data to inform clinical care.

Working alongside the clinical team in MBC helped the evaluation team recognize when different families required different supports to complete PROMs and identify the best ways to support them. Support options included meeting with parents/caregivers during visits while the client received care or completing measures together remotely (phone/videoconference), sometimes with support from an interpreter. Although this could add time to the visit, it was vital that families felt supported when completing the PROMs. Donelan et al. (2025) describe a similar situation in which a caregiver became disengaged from the program due to the amount of contact and information from the team. By collaborating with this parent, they were able to identify strategies that made engagement easier, such as limiting the number of treatment suggestions shared at one time (65).

Families entering the ENS program are facing significant unmet needs, which contribute to considerable stress and overwhelm. In this context, the task of completing measures can feel burdensome, potentially contributing to lower completion rates. Furthermore, some caregivers may face challenges related to cognitive capacity, literacy, or language barriers, highlighting the need for appropriate accommodations such as simplified materials or translation services to support equitable participation. Barron et al. identified appropriate selection of PROMs as a key strategy to improve completion rates (66). Our original battery included two longer measures, the ABAS-3 and the BASC-3. The ABAS-3 was subsequently removed based on feedback from clinicians and families about the burden of completing this measure. BASC-3 completion rates remain lower than those for other measures; for this reason, we are investigating whether a shorter measure can provide similar information and demonstrate sensitivity to change. Future evaluations of this population can include biomarkers, such as neuroimaging and EEG data, alongside PROMs, and may also apply advanced analytic approaches.

## 5 Limitations

Our implementation is not without shortcomings. Due to the need for timely identification of measures, we did not conduct a systematic review or a review of the gray literature. As such, we may have missed relevant programs or measures. As noted above, consultation with family caregivers and youth was deferred until we had a clearer understanding of the types of children and families who would be served by the program. This paper describes our implementation; program-wide clinical outcomes will be described in a forthcoming paper.

## 6 Conclusions

We implemented MBC in a novel, needs-based program by considering program aims, examining existing literature, and soliciting repeated input from clinicians. MBC will enable data-driven decisions for individual clients, program sites, and the multi-site program. Consideration of measurement burden and integration into clinical processes is key to improving PROM completion. Future evaluation will focus on sustainability, clinician buy-in, feedback utilization, and the relationship between MBC uptake and clinical outcomes.

## Data Availability

The original contributions presented in the study are included in the article/supplementary material. Further inquiries can be directed to the corresponding authors.

## Appendix 1

**Table:** Full list of potential measures.

Measure	Concept/Domain	Citation	Population	Format
About My Child	Complexity	Rosenbaum, P., Mesterman, R., Law, M., Jaffer, S., Russell, D., Gorter, J. W., McCauley, D. & Kertoy, M. (2008) *About My Child, 26-Item Version*. *CanChild* Centre for Childhood Disability Research, Hamilton, ON.	Any child 0-18, not specific to diagnosis	26 items, caregiver report; includes open ended comment section for additional information
PROMIS Pediatric Global Health Measure	Complexity	Forrest et al. (25)	8–17 years old or parents of children 5-17	7-item self-report
Aberrant Behaviour Checklist	Complexity (Interfering Behaviours)	Aman et al. (24)	Individuals with IDD, 5-51+	5 subscales, 58 items, each item is rated 0-3
Behavior Assessment System for Children (BASC-3)	Complexity (Interfering Behaviours)	Reynolds, C.R. & Kamphaus, R.W. (23).	Ages 6-21	PRS-child, ages 6-11–175 items (rating scale) PRS-adolescent, ages 12-21, - 173 items (rating scale) SRP-child, ages 8-11–137 items (rating scale)_ SRP-adolescent, ages 12-21–189 items (rating scale)
The Behaviour Problems Inventory	Complexity (Interfering Behaviours)	Rojahn et al. (29)	Ages 14-91	52 items respondent-based behaviour rating instrument (severity and frequency scale)
Child Behaviour Checklist (CBCL)	Complexity (Interfering Behaviours)	Achenbach, T. M., & Rescorla, L. A. (22)	Ages 1.5 – 18	Ages 1.5-5–78 items (rating scale) Ages 6-18–83 items (rating scale) + some fill in the blank with ratings Ages 11-18 (youth self-report) - 112 items (rating scale) + some fill in the blank with ratings
Child’s Challenging Behaviour Scale	Complexity (Interfering Behaviours)	Bourke-Taylor et al. (30)	Mothers of school age children with disability	11 item scale
Repetitive behaviour Scale-Revised	Complexity (Interfering Behaviours)	Bodfish et al. (31)	Autistic children, adolescents, adults	44 item guardian/self-report, 4.0 scale quantifying how much of a problem behaviour has been over past month
Selective Mutism Questionnaire	Complexity (Interfering Behaviours)	Bergman et al. (32)	Child ages 3-11	Parent report, 17 items
Strengths and Weaknesses of Attention-Deficit/Hyperactivity and Normal Behaviour Scale	Complexity (Interfering Behaviours)	Swanson et al. (33)	Parent report for children and adolescents (6-17)	Parents rate behaviours in variety of settings over 12 months on 7-point scale
Health Status Questionnaire	Complexity (Medical) + High intensity users	Health Status Questionnaire 2.0 (HSQ 2.0) (2002). PsycTESTS. APA.		45 question survey
Medical Status Questionnaire	Complexity (Medical)	Newton-Wellesley Ambulatory Care Center		
Pediatric Complex Chronic Conditions	Complexity (Medical)	Feudtner et al. (34)		
The Autism-Tics, ADHD and other Comorbidities inventory (A-TAC)	Complexity (Mental Health)	Marland et al. (35)	Children with neurodevelopmental disorders	
Mini International Neuropsychiatric Interview for Children and Adolescents	Complexity (Mental health)	Sheehan et al. (36)	Children ages 6–17 years	Short, structures, diagnostic interview (15 minutes to administer)
Mood and Feelings Questionnaire	Complexity (Mental health)	Angold & Costello (37)	Ages 6-19	Self or parent report,
The Revised Child Anxiety and Depression Scale	Complexity (Mental Health)	Chorpita & Spence	Youth age 8-18, can be completed by parent or caregiver	47-item self-report
Screen for Child Anxiety Related Emotional Disorders	Complexity (Mental Health)	Birmaher et al. (38)	Children ages 8-18	Self or parent report, 41 items
Spence Child Anxiety Scale	Complexity (Mental Health)	Spence (39)	8 to 15 years old	44 items (6 filler), scored on 4-point scale to determine frequency
Adaptive Behaviour Assessment System	Daily Living Skills	Harrison & Oakland (40)	Normative data across lifespan	Behavior rating scale typically completed by parent, caregiver, and/or teacher; self-rating option for adults: Parent/Primary Caregiver Form (Ages 0–5), Teacher/Daycare Provider Form (Ages 2–5), Parent Form (Ages 5–21), Teacher Form (Ages 5–21), Adult Form (Ages 16–89): takes 15–20 minutes to complete per form
Vineland Adaptive Behaviour Scales	Daily Living Skills	Sparrow, Cicchetti, & Balla (41)	Birth to age 90	Can be administered as interview or as self/parent report
Brief Family Assessment Measure	Fragility (of family)	Skinner et al., 1995 (42)	Normative data for typical adults and typical adolescents	
The Brief Family Distress Scale	Fragility (of family)	Weiss & Lunksy, 2011 (27)	Caregivers of people with ASD	Continuum of distress, 10-point scale to rate family crisis
The Family Empowerment Scale	Fragility (of family)	Koren et al., (43)	Parents of children with emotional disabilities	Self-report, 28 items, 5-point likert
Family Hardiness Index	Fragility (of family)	McCubbin et al., (44)	?	20 items
Inventory of Family Protective Factors	Fragilty (of family)	Gardner et al. (45)	?	Self-report, 16 items scored on 5-likert
Los Angeles Epidemiologic Catchment Area (LA-ECA) Survey	High-intensity users	United States Department of Health and Human Services (46)	18+ years, general population	
Medical Expenditure Panel Survey	High intensity users	Zuvekas & Olin (47)	Civilian population	Variable length depending on survey, self-reported
Resource Use Questionnaire	High intensity users	Ungar et al. (28)	RUQ-A: 11-18 RUQ-P: preschool age RUQ-T: NDD/Autism	
Questionnaire for Measuring Health-Related Quality of Life in Children and Adolescents (KINDL)	Quality of life	Ravens-Sieberer, U., & Bullinger, M. (48)	Ages 4-17	Child/Teen Self-report Ages 4-6–19 items (rating scale) – interview Ages 7-13 & Ages 14-17–31 items (rating scale) Parent/Caregiver Report Ages 3-6 & Ages 7-17–24 items (rating scale)
Canadian Occupational Performance Measure	Symptom Improvement/Behaviour Change	Law et al. (49)	All ages (Including children)	Semi-structured interview
CDC Milestone Checklist	Symptom Improvement/Behaviour Change	Centers for Disease Control and Prevention (CDC) (50)	Parents can use for children up to 5 years of age	List divided into social/emotional, language/communication, cognitive, and physical milestones
Goal Attainment Scale	Symptom Improvement/Behaviour Change	Kiresuk, T. J., & Sherman, R. E. (51)	Preschool, child and adolescent versions	Involves identifying and weighing specific goals, defining expected outcomes and rating goal attainment at a review date (allows for longitudinal follow up with sensitivity to goals built in)
National Survey of Children’s Health	Unmet needs	United States Census Bureau (52)	Children 0-17	
Pennsylvania Autism Needs Assessment survey	Unmet needs	ASERT bringing autism resources together (53)	Caregiver and individual survey; caregiver can be completed for any age	13 pages, combination of check boxes/fill in the blank and short answer
